# Integrating predicted gene expression into genomic selection: an evaluation in partridge chickens under realistic breeding scenarios

**DOI:** 10.1186/s12711-026-01099-w

**Published:** 2026-11-09

**Authors:** Huimin Kang, Weicong Wu, Zheng Ma, Tingting Yang, Hai Xiang, Fei Ye, Hui Yu, Hua Li

**Affiliations:** 1 Guangdong Provincial Key Laboratory of Animal Molecular Design and Precise Breeding, School of Animal Science and Technology, Foshan University, Foshan, 528000 China; 2 Guangdong Tinoo’s Co. Ltd, Jiangkou, Feilaixia, Qingcheng, Qingyuan, Guangdong 511827 China

## Abstract

**Background:**

Genomic prediction (GP) has enhanced breeding value prediction using genome-wide markers and genotype–phenotype associations. Integrating transcriptomic data, which are closer to phenotypes, may improve breeding value prediction accuracy. While multi-omics integration in livestock is cost-prohibitive, resources such as FarmGTEx enable large-scale SNP-based prediction of estimated breeding values (EBVs) for gene expression. However, utility of these predicted EBVs for enhancing GP accuracy remains underexplored. We evaluated the integration of observed gene expression and gene expression EBVs into genomic prediction models for live, dressed, and eviscerated weights in 350 Tiannong Partridge Chickens. Whole-genome resequencing and pectoral muscle transcriptomes were generated, and multi-omics models were assessed across three scenarios: (I) both reference and validation populations have transcriptomic data; (II) only the reference population has transcriptomic data; (III) neither has transcriptomic data—requiring cross-population prediction using the ChickenGTEx reference panel. We compared genomic–transcriptomic best linear unbiased prediction (GTBLUP) models incorporating gene expression EBVs predicted via best linear unbiased prediction (BLUP) or elastic net (ENET) (GTBLUP_pred_BLUP and GTBLUP_pred_ENET, respectively), genomic-feature BLUP (GFBLUP) based on expression quantitative trait loci, and standard genomic BLUP (GBLUP). Model prediction accuracies were compared using *t*-tests followed by Holm correction.

**Results:**

In Scenario I, integrating observed expression data significantly improved phenotype prediction accuracy over GBLUP (adjusted *p* < 0.05; reference-only). Subsequent comparisons are genomic prediction accuracy. For Scenario II, the GTBLUP_pred_ENET model (using genes with *h*^2^ > 0.7) showed modest numerical gains over GBLUP when using gene expression EBVs of the validation population, although the differences were not statistically significant (adjusted *p* > 0.05). Furthermore, using the cross-population ChickenGTEx reference panel for expression EBV prediction (Scenario III), GTBLUP_pred_ENET (*h*^2^ > 0.3) again yielded consistently higher prediction accuracies than GBLUP, albeit without statistical significance (adjusted *p* > 0.05). In contrast, GFBLUP showed minimal or negative gains, especially when regulatory signals were extrapolated from external populations. For expression EBV prediction, ENET generally outperformed BLUP in Scenarios II and III. We found a low-to-moderate positive correlation between gene expression prediction accuracy and its cis-heritability, although overly stringent heritability filtering reduced genomic prediction accuracy.

**Conclusions:**

These results highlight the potential of predicted expression EBVs for GP, pending validation in larger, more diverse populations and traits, alongside methodological refinements.

**Supplementary Information:**

The online version contains supplementary material available at https://doi.org/10.1186/s12711-026-01099-w.

## Background

Livestock and poultry breeding form the cornerstone of modern agricultural systems, playing a pivotal role in ensuring global food security and advancing sustainable agricultural development. With rising global population and increasing pressures from climate change, the demand for high-quality animal protein continues to grow. Conventional breeding strategies—relying primarily on phenotypic records and pedigree information—are increasingly constrained by their limited accuracy and long generation intervals, rendering them insufficient to meet current and future production challenges. Genomic prediction (GP), which leverages high-density molecular markers across the genome, has emerged as a transformative approach to accelerate genetic progress.

First proposed by Meuwissen et al. in 2001, GP utilizes genome-wide single nucleotide polymorphisms (SNPs) to estimate genomic estimated breeding values (GEBVs) [1]. Unlike traditional marker-assisted selection (MAS), GP does not require prior knowledge of quantitative trait loci (QTL) and can simultaneously capture the collective effects of numerous loci with small individual contributions [2]. This enables more accurate prediction of breeding values. As a result, GP has been widely adopted in major livestock industries—including dairy and beef cattle, pigs, and poultry—delivering substantial improvements in genetic gain and economic efficiency [3, 4].

The predictive performance of GP hinges critically on the statistical models employed. Among these, genomic best linear unbiased prediction (GBLUP) and its single-step extension (ssGBLUP) are the most widely used in animal breeding. These methods operate within a mixed-model framework and employ a genomic relationship matrix to replace the traditional pedigree-based one, thereby enhancing the accuracy of genetic evaluations [5, 6]. However, these models primarily rely on genomic markers and largely ignore the increasingly abundant multi-omics data now available.

Multi-omics layers serve as functional intermediates between genotype and phenotype [7]; indeed, many genetic variants exert their influence on complex traits indirectly through regulatory effects on gene expression or metabolic pathways [8]. Notably, transcriptomic profiles reside closer to the phenotypic outcome in the central dogma of molecular biology [9]. This biological insight provides a strong rationale for incorporating transcriptomic information into GP models. Evidence from studies in humans, plants, and animals suggests that integrating multi-omics data can enhance the accuracy of phenotype and GEBV prediction [10–12]. Current integration strategies fall broadly into three categories: (1) direct integration for phenotype prediction, where omics-derived variables (e.g., gene expression levels) are used directly as predictors in models such as transcriptomic best linear unbiased prediction (TBLUP) [13] or integrated genomic-transcriptomic best linear unbiased prediction (GTBLUP) [12]; (2) feature selection for GEBV prediction, in which SNPs associated with molecular phenotypes—identified via expression quantitative trait locus (eQTL) mapping—are incorporated as distinct effect terms in GP models [14]; and (3) intermediary-trait integration for GEBV prediction, in which omics-derived variables are incorporated as intermediary traits into genetic evaluation models to predict genetic effects [15, 16].

Despite their potential, large-scale application of multi-omics data in routine breeding programs remains hindered by high sequencing costs. Consequently, only a limited number of individuals typically have omics-level measurements, while the majority are characterized solely by genotypic and phenotypic data. A critical breakthrough in overcoming this barrier lies in recognizing that gene expression itself is a heritable trait, influenced by underlying genetic variation [17, 18]. This allows gene expression to be modeled as a “complex quantitative trait”, enabling the development of predictive models that estimate genetically regulated expression (GReX) from SNP genotypes alone [19]. Such models are trained on reference populations with both genomic and transcriptomic data and can then be applied to impute GReX for thousands of individuals based solely on their DNA sequences [20, 21]. This paradigm has been successfully implemented in human genetics, particularly in transcriptome-wide association studies (TWAS), where imputed GReX plays a central role in identifying candidate genes associated with complex diseases and traits [22, 23].

This concept has recently been extended to livestock through initiatives such as the FarmGTEx project, which aims to characterize the genetic regulation of gene expression across 16 domesticated species under diverse biological and environmental conditions [24]. To date, comprehensive genotype-tissue expression (GTEx) atlases have been established for key agricultural species, including chicken (Gallus gallus) [25], pig (Sus scrofa) [26], and cattle (Bos taurus) [27]. These resources provide a foundation for predicting estimated breeding values (EBVs) for tissue-specific gene expression from genotypes, opening new possibilities for enhancing GP through the integration of these predicted EBVs. Nevertheless, the utility of predicted expression EBVs for improving genomic prediction accuracy in livestock breeding remains largely unexplored.

In this study, we investigate the feasibility of improving genomic prediction performance in Tiannong Partridge Chickens by integrating transcriptomic information. We evaluate the predictive performance of GP models using both experimentally sequenced and predicted gene expression data—latterly derived from the FarmGTEx reference panel. Furthermore, we compare these direct integration approaches with a feature selection strategy that incorporates eQTL-informed genomic features into the prediction model. Our findings provide insights into the potential of multi-omics data to improve genetic evaluation in poultry breeding programs.

## Materials and methods

### Tiannong partridge chicken dataset

#### Animals and phenotypic measurements

A total of 350 healthy female Tiannong partridge chickens were sourced from Guangdong Tinoo’s Co., Ltd. The Tiannong partridge chicken is a commercially used three-way cross chicken bred from three pure lines of the Qingyuan partridge chicken, a prominent indigenous Chinese slow-growing yellow-feathered broiler breed valued for its superior meat quality. The birds were randomly sampled from a single hatching cohort. From day 1 to day 125, they were raised under standardized management conditions at one of the company’s commercial farms. All birds were group-housed in a single open-concept facility and maintained under identical environmental, husbandry, and feeding protocols throughout the rearing period. On day 126, following a 12-hour feed withdrawal period, all birds were processed on the same day using a mechanized slaughter line to minimize procedural variability. Three production traits—live weight (LW), dressed weight (DW), and eviscerated weight (EW)—were measured according to the Terminology and Measurement Statistics for Poultry Production Performance (NY/T823-2020), and used in downstream analyses.

#### Genotypic data processing

Genomic DNA was extracted from blood samples collected from each individual chicken. Whole-genome resequencing was performed with an average sequencing depth of 4× per sample. Raw sequencing reads underwent quality control using Trimmomatic [28] with default parameters, and high-quality paired-end reads were retained for further analysis. Cleaned reads were aligned to the chicken reference genome [GRCg6a (GCA_000002315.5)] using the MEM algorithm implemented in Burrows-Wheeler Aligner (BWA) [29]. Resulting SAM files were converted to BAM format, sorted, and indexed using Samtools [30]. PCR duplicates were removed using PICARD (https://broadinstitute.github.io/picard/).

Joint SNP calling across all samples was conducted using the GATK Best Practices pipeline, including the HaplotypeCaller, CombineGVCFs, and GenotypeGVCFs modules, to generate a multi-sample VCF file [31]. Variant filtration was performed using the VariantFiltration module in GATK with the following criteria: QD > 2.0, FS < 60.0, MQ > 40.0, MQRankSum > −12.5, and ReadPosRankSum > −8.0. Missing genotypes were imputed using Beagle v5.4 [32]. Subsequent quality control of SNP data was carried out using PLINK v1.9 [33] with the following filters: SNP call rate ≥ 95%, minor allele frequency (MAF) ≥5%, Hardy-Weinberg equilibrium *p*-value ≥ 1.0 × 10^−6^, individual call rate ≥ 95%, and exclusion of SNPs located on sex chromosomes. After quality control (QC), 2,138,157 autosomal SNPs from 350 individuals were retained for downstream analyses.

To ensure comparability across all subsequent models, we used the identical set of QC-passed autosomal SNPs without linkage disequilibrium (LD) pruning. This choice was empirically justified: systematic LD pruning—performed using PLINK v1.9 (–indep-pairwise 500 50 0.1, 0.5, and 0.9)—consistently reduced prediction accuracy under 10 independent repetitions of five-fold cross-validation in preliminary GBLUP analyses (Additional file 1 Fig. S1). Therefore, given that pruning discards potentially informative markers without improving prediction accuracy, we retained the full unpruned SNP set as the common genomic input for all downstream models.

#### Transcriptomic data processing

During slaughter, right pectoral muscle tissue samples were collected from identical anatomical positions across all individuals. Tissues were immediately snap-frozen in liquid nitrogen and stored at −80 °C until RNA extraction. Total RNA was isolated using TRIzol reagent (Invitrogen, Carlsbad, CA, USA), and RNA quality was assessed using a Kaiao K5500® spectrophotometer (Kaiao, Beijing, China) for purity (A₂₆₀/A₂₈₀ ratio between 1.8 and 2.0), and an Agilent Bioanalyzer 2100 system with RNA Nano 6000 kit (Agilent Technologies, Santa Clara, CA, USA) for integrity (RIN > 7.5).

cDNA libraries were constructed following standard protocols: mRNA was enriched using oligo(dT)-coupled magnetic beads, followed by fragmentation and synthesis of double-stranded cDNA. The resulting cDNA was purified using the QIAQuick PCR Purification Kit (QIAGEN, Valencia, CA, USA), then subjected to end repair, A-tailing, adapter ligation, size selection, and PCR amplification. Library quality was validated prior to sequencing. Paired-end 150 bp reads were generated on an Illumina NovaSeq 6000 platform (Illumina, San Diego, CA, USA).

Raw transcriptomic reads were processed as follows: FastQC was used for initial quality assessment; adapters were trimmed using BBMap [34], and low-quality reads were filtered using Fastp. Cleaned reads were aligned to the chicken reference genome (GRCg6a) using HISAT2 [35]. Aligned reads in SAM format were converted to BAM files using Samtools. Gene-level counts were quantified using FeatureCounts [36], and gene expression levels were normalized via DESeq2 [37]. Final expression matrices included 12,691 genes across 350 individuals.

### ChickenGtex project dataset

Muscle transcriptomic data and genotypic data from 517 chickens across multiple populations were obtained from the FarmGTEx Chicken Project [25] (ChickenGTEx; http://chickengtex.farmgtex.org/), comprising gene expression profiles for 12,477 genes and 1,522,091 SNPs. The same reference genome (GRCg6a) was used for consistency in alignment and analysis. To enable cross-population integration and ensure SNP compatibility, only variants shared between the ChickenGTEx and Tiannong datasets were retained, yielding a set of 289,730 common SNPs. These overlapping SNPs were used in subsequent gene expression EBV prediction, with the ChickenGTEx dataset serving as the training population.

### Scenario design for multi-omics genomic selection

**Fig. 1:**
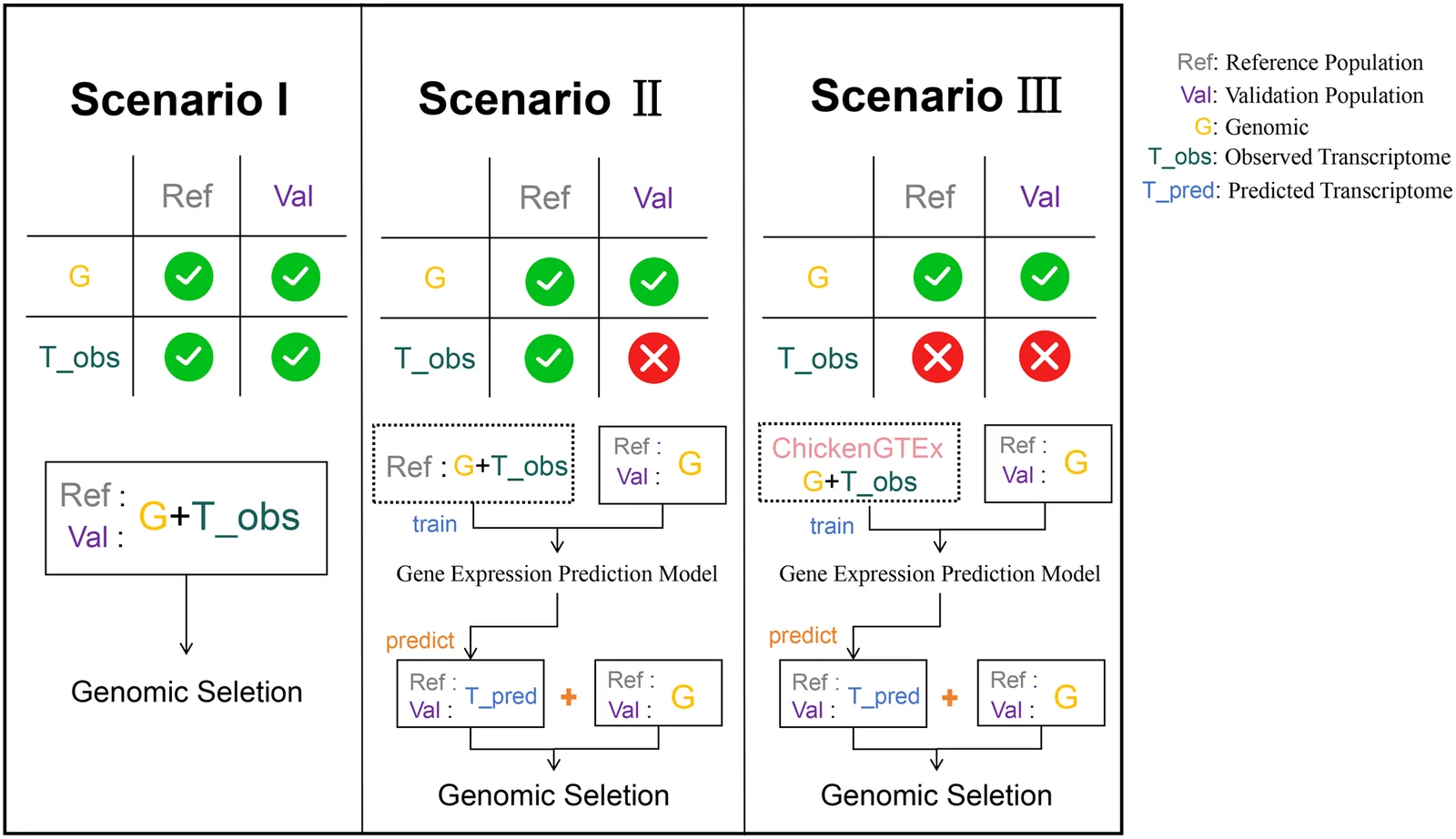
Schematic illustration of the three multi-omics genomic prediction validation scenarios (I–III)

To evaluate the impact of integrating transcriptomic data into genomic prediction, three distinct scenarios were designed (Fig. 1). In all scenarios, both the reference and validation populations had genotype data. In Scenario I, observed transcriptomic data were available for both populations. In Scenario II, only the reference population had observed transcriptomic data, and gene expression EBVs for the validation population were predicted using models trained on the reference set. In Scenario III, neither population had transcriptomic data; instead, a cross-population prediction strategy was employed, in which the ChickenGTEx dataset served as the training set for gene expression EBV prediction. To determine how best to leverage these predicted gene expression EBVs in genomic prediction, two downstream integration strategies were compared: (1) inclusion of predicted expression EBVs for all successfully modeled genes, and (2) inclusion of genes whose heritability exceeded predefined thresholds (0.1, 0.3, 0.5, and 0.7).

Prediction accuracy was assessed using a five-fold cross-validation design, repeated ten times independently to ensure robust estimation, with the same fold assignments employed across the three scenarios. Given the absence of pedigree information and to minimize potential bias in genomic prediction accuracy arising from cryptic genetic relationships between individuals in the training and validation sets, a genomic relationship matrix was constructed using genome-wide SNP data to evaluate pairwise genetic relatedness among individuals. The genomic relationship matrix revealed a maximum relationship coefficient of 0.34 across the population, with a mean of −0.003 ± 0.014, indicating negligible levels of close kinship (e.g., no full-sib pairs). This confirms that the population is suitable for unbiased evaluation of prediction performance. Prediction accuracy was defined as the Pearson correlation coefficient between observed phenotypes and EBVs in the validation population, with final results reported as the mean across replicates.

To statistically compare the prediction accuracies among different multi-omics models and the baseline method using genomic data only within each scenario, we performed pairwise *t*-tests using the accuracies obtained from the ten independent 5-fold cross-validation replicates. Because repeated cross-validation estimates are non-independent due to overlapping training and test sets across folds, we applied the corrected dependent *t*-test using the repkfold_ttest() function from the correctR package [38] to account for the covariance structure induced by sample reuse. All tests were two-sided, with the null hypothesis ${H_0}: {\mu _1} = {\mu _2}$. To control the family-wise error rate (FWER) across all pairwise comparisons performed within each scenario, we applied the Holm–Bonferroni step-down procedure [39] separately for each scenario. Adjusted *p*-values were considered statistically significant at the threshold of *p* < 0.05.

### Estimation of cis-heritability of gene expression

Gene expression can be treated as a heritable quantitative trait influenced by genetic variation [17]. Expression heritability quantifies the proportion of variance in transcript levels attributable to genetic factors [40]. Previous studies indicate that incorporating gene expressions with high prediction accuracy—and by extension, high heritability—into genomic prediction models improves prediction performance for breeding value prediction [18, 41].

The model used for cis-heritability estimation is:

1 $${\bf{y}} = {\bf{X}\boldsymbol{\alpha}} + {\bf{Z}}{{\bf{g}}_{a - cis}} + {\bf{e}},$$

where ${\bf{y}}$ is the vector of standardized molecular phenotypes; ${\bf{X}}$ is the matrix of covariates, ${\bf{\alpha }}$ is the vector of covariate regression coefficients; ${{\bf{g}}_{a - cis}}$ is the vector of additive genetic effects with ${{\bf{g}}_{a - cis}} \sim N\left( {\bf{0},{{\bf{A}}_c}\sigma _{a - cis}^2} \right)$, where ${{\bf{A}}_c}$ is an additive genomic relationship matrix constructed from cis-SNPs (defined as SNPs located within ±1 Mb of the transcription start site of the corresponding gene) using VanRaden’s Method 1 [6], and **Z** is the corresponding design matrix; and ${\bf{e}}$ is the *n × 1* vector of residuals with ${\bf{e}} \sim N\left( {0,{\bf{I}}\sigma _{\rm{e}}^2} \right)$.

More specifically, covariates in the model include probabilistic estimation of expression residuals (PEER) factors derived from transcriptomic data and principal components (PCs) derived from genomic data. PEER is a Bayesian framework based on factor analysis that enables the inference of latent covariates from expression data, and these factors typically correspond to unrecorded experimental batches, RNA quality variations, and other technical noise. PEER factors were estimated using the PEER method as implemented in the PEER R package [42]. The composition of covariates varied depending on the training dataset. In the models for Tiannong partridge chickens, a single PEER factor was included as a covariate, and principal components were not included because there was no detectable population stratification. In models for the ChickenGTEx dataset, 60 PEER factors and the first five genomic PCs were incorporated, and these genomic PCs were obtained from the ChickenGTEx project. The optimal number of PEER factors was determined based on their contribution to genomic prediction accuracy in preliminary evaluations.

We estimated cis-heritability using the OmiGA1.0.0.2 software package [43]. Cis-heritability of gene expression was then estimated separately for each scenario. In Scenario II, heritability estimates were computed individually for each fold and repetition.

### Models for predicting gene expression estimated breeding values and prediction accuracy assessment

In Scenarios II and III, two statistical approaches—linear unbiased prediction (BLUP) and elastic net (ENET)—were employed to predict gene expression EBVs using cis-SNPs located within ±1 Mb of the transcription start site [26]. The same covariates and SNPs were used as for estimation of gene expression cis-heritability.

#### Best linear unbiased prediction model

The linear mixed model used to predict breeding values for gene expression is:

2 $${\bf{t}} = {\bf{Xb}} + {\bf{Z}}{{\bf{g}}_{a - cis}} + {\bf{e}},$$

where ${\bf{t}}$ is the vector of observed gene expression levels for a given gene; ${\bf{b}}$ represents fixed regression coefficients of PEER factors and PCs; ${{\bf{g}}_{a - cis}}$ is the vector of random genetic effects assumed to follow $N\left( {0,{{\bf{A}}_c}\sigma _g^2} \right)$, with ${{\bf{A}}_c}$ being the additive genomic relationship matrix constructed from cis-SNPs; ${\bf{X}}$ and ${\bf{Z}}$ are the design matrices; and ${\bf{e}}$ is the residual vector distributed as $N\left( {0,{\bf{I}}\sigma _e^2} \right)$.

The additive genomic relationship matrix ${{\bf{A}}_c}$ was calculated as [6]:

3 $${{\mathbf{A}}_c}{\mkern 1mu} = {\mkern 1mu} \frac{{{\mathbf{M}}{{\mathbf{M}}^\prime }}}{{2\sum\nolimits_{i = 1}^m {{p_i}(1 - {p_i})} }},$$

where ${\bf{M}}$ is the centered genotype matrix of cis-SNPs and ${p_i}$ denotes the minor allele frequency of the *i*th SNP.

#### Elastic net model

A penalized linear regression model was also formulated to predict breeding values for gene expression, as follows:

4 $${\bf{t}} = {\bf{X}\boldsymbol{\beta }} + {\bf{e}},$$

where ${\bf{\beta }}$includes regression coefficients for PEER factors, PCs, and cis-SNP genotypes.

The ENET estimator minimizes the objective function:

5 $$\widehat {{\beta }} = \mathop {min}\limits_{{\beta }} \,\left\{ {\frac{1}{{2N}}\left\| {\bf{t}} \right. - \left. {{{\bf{X}\boldsymbol{\beta }}}} \right\|_2^2} \right. + \left. {\lambda \left( {\alpha \left\| {{{\left. {\boldsymbol{\beta }} \right\|}_1}} \right. + \frac{{\left( {1 - \alpha } \right)}}{2}\left\| {\left. {\boldsymbol{\beta }} \right\|} \right._2^2} \right)} \right\},$$

where *N* is the sample size, *λ * controls overall regularization strength, and *α * determines the balance between L_1_ (lasso) and L_2_ (ridge) penalties. In this study, *α * was fixed at 0.5, and *λ * was optimized via 10-fold cross-validation using the R package glmnet [44].

For BLUP, all genes were successfully fitted. For ENET, genes were considered successfully fitted only when the glmnet function returned a fitted model and predicted expression EBVs; genes without valid output were excluded from downstream analyses.

Prediction accuracy of gene expression EBVs was assessed as the Spearman correlation (*R*) between predicted and observed gene expression levels, and then divided by the square root of the heritability of gene expression. In Scenario II, prediction models were built independently for each fold in every cross-validation repetition. Thus, final accuracy reflects the average across five folds and ten replicates. For Scenario III, accuracy of predicted gene expression EBVs was calculated only for genes common to three sets: (1) genes with successfully fitted prediction models, (2) genes present in the Tiannong Partridge Chicken expression dataset, and (3) genes included in the ChickenGTEx expression dataset.

### Statistical models for multi-omics genomic prediction

We applied three multi-omics genomic prediction models—GTBLUP, transcriptomic-genomic BLUP (TGBLUP), and genomic-feature BLUP (GFBLUP). Specifically, we used GTBLUP and TGBLUP in Scenario I, and GTBLUP and GFBLUP in Scenarios II and III. For comparison, we also implemented the standard GBLUP model, which is based solely on genomic data. Given this homogeneous experimental design—in which batch, sex, facility, housing, management, feeding regimen, health management, and processing date were identical for all birds—the potential systematic environmental factors had no among-individual variation. They therefore could not be included as fixed effects in the genomic prediction models, and their significance could not be tested. Accordingly, no correction for these factors was applied to the phenotypic records, and raw phenotypes were used directly for genomic prediction.

#### Genomic best linear unbiased prediction

The basic GBLUP model is:

6 $${\bf{y}} = \mu \bf{1} + {{\bf{Z}}_g}{\bf{g}} + {\bf{e}}$$

where ${\bf{y}}$ is the vector of phenotypic observations; *μ *is the overall mean; ${\mathbf{g}} \sim N\left( {0,{\mathbf{G}}\sigma _g^2} \right)$ represents additive genetic effects; and ${\mathbf{e}} \sim N\left( {0,{\mathbf{I}}\sigma _e^2} \right)$ is the residual term. This model served as the baseline for comparison.

#### Genomic-transcriptomic best linear unbiased prediction

The GTBLUP model incorporates EBVs for gene expression into genomic prediction:

7 $${\bf{y}} = \mu \bf{1} + {{\bf{Z}}_g}{\bf{g}} + {{\bf{Z}}_{{t_g}}}{{\bf{t}}_g} + {\bf{e}},$$

where ${{\bf{t}}_g}$ is the vector of partial EBVs for the trait based on EBVs for gene expression, and is assumed to follow $N\left( {0,{{\bf{T}}_g}\sigma _{{t_g}}^2} \right)$, and ${{\bf{t}}_g}$ is the relationship matrix among gene expression EBVs, computed as:

8 $${{\bf{T}}_g} = {{{\bf{W}}{{\bf{W}}^\prime}} \over k},$$

with ${\bf{W}}$ denoting the standardized and centered matrix of gene expression EBVs and *k* the number of genes. The ${{\bf{t}}_g}$ term is equivalent to fitting the expression EBV for each gene as a random covariate with equal variance across genes, analogous to the equivalence between GBLUP and the model based on random regressions on marker covariates. The genomic relationship matrix ${\bf{G}}$ was constructed using all SNPs.

In Scenario I, we applied GTBLUP using observed gene expression data and denote this approach as GTBLUP_obs. In Scenarios II and III, we applied GTBLUP using predicted expression EBVs generated by BLUP or by ENET; the corresponding methods are termed GTBLUP_pred_BLUP and GTBLUP_pred_ENET, respectively. Submodels that further filtered genes based on cis-heritability were labeled with suffixes indicating the heritability threshold: GTBLUP_pred_BLUP_0.1, GTBLUP_pred_BLUP_0.3, GTBLUP_pred_BLUP_0.5, and GTBLUP_pred_BLUP_0.7, where only genes with heritability above the given threshold were retained. For comparison, the model using all predicted gene expression EBVs without filtering was denoted as GTBLUP_pred_BLUP_all. An analogous naming scheme applied to the GTBLUP_pred_ENET variants. Importantly, GTBLUP_obs was used for phenotype prediction in Scenario I, whereas GTBLUP_pred_BLUP and GTBLUP_pred_ENET were used for GEBV prediction in Scenarios II and III. Therefore, the prediction accuracies of GTBLUP_obs and the other models are not directly comparable.

For each scenario, the optimal variant within each method family was selected based on maximal prediction accuracy: among the GTBLUP_pred_BLUP variants (GTBLUP_pred_BLUP_all, GTBLUP_pred_BLUP_0.1, GTBLUP_pred_BLUP_0.3, GTBLUP_pred_BLUP_0.5, and GTBLUP_pred_BLUP_0.7), the one achieving the highest cross-validated prediction accuracy was designated GTBLUP_pred_BLUP_best; similarly, the top-performing variant among the GTBLUP_pred_ENET series was labeled GTBLUP_pred_ENET_best. These two best-performing models were then benchmarked against all other competing methods under identical evaluation conditions.

#### Transcriptomic-genomic best linear unbiased prediction

TGBLUP is adapted from the MGBLUP [45] framework of Christensen et al. [15], substituting metabolomic with transcriptomic data. It involves solving two sequential linear mixed models and is applicable only in Scenario I, where observed gene expressions are available.

First step:

9 $${\bf{y}} = \mu \bf{1} + {{\bf{Z}}_g}{{\bf{g}}_1} + {{\bf{Z}}_{{t_o}}}{{\bf{t}}_o} + {{\bf{e}}_1},$$

where ${{\bf{g}}_1}{\rm{ }}$is the vector of additive genomic effects based on all SNPs, assumed to follow $N\left( {0,{\bf{G}}\sigma _{{g_1}}^2} \right)$; ${{\bf{t}}_o}$ is the vector of observed gene expression effects, assumed ${{\bf{t}}_o} \sim N\left( {0,{{\bf{T}}_o}\sigma _{{t_o}}^2} \right)$, with ${{\bf{t}}_o}$ computed analogously to ${{\bf{T}}_g}$ but using observed expression data; and ${{\mathbf{e}}_1} \sim N\left( {0,{\mathbf{I}}\sigma _{{e_1}}^2} \right)$ is the residual term.

Second step:

10 $${{\bf{t}}_o} = \mu \bf{1} + {{\bf{Z}}_g}{{\bf{g}}_2} + {{\bf{e}}_2},$$

where ${{\bf{g}}_2}$ represents genetic effects on gene expression, assumed to follow $N\left( {0,{\bf{G}}\sigma _{{g_2}}^2} \right)$; and ${{\mathbf{e}}_2} \sim N\left( {0,{\mathbf{I}}\sigma _{{e_2}}^2} \right)$ is the residual term.

The total breeding value is defined as ${\bf{a}} = {{\bf{g}}_1} + {{\bf{g}}_2}$.

#### Genomic-feature best linear unbiased prediction

GFBLUP was applied in both Scenarios II and III. In GFBLUP, SNPs are partitioned into two classes: feature SNPs (selected based on eQTL signals) and remaining SNPs [46]. In the eQTL analysis, cis-SNPs were defined as SNPs located within ±1 Mb of the transcription start site of the target gene, consistent with the definition used in the model for predicting breeding values for gene expression, whereas trans-SNPs were defined as SNPs located outside this ±1 Mb region of the target gene. Univariate association tests between SNPs and gene expression levels were conducted using a mixed model in OmiGA1.0.0.2 [43], adjusting for the same covariates used for gene expression EBV prediction. Significance was assessed using the clipper method [47] for FDR correction, with feature SNPs defined at a 5% FDR threshold [25]. In Scenario III, eQTL analysis was restricted to the 289,730 common SNPs between datasets.

The GFBLUP model is:

11 $${\bf{y}} = \mu 1 + {{\bf{Z}}_{{g_f}}}{{\bf{g}}_f} + {{\bf{Z}}_{{g_r}}}{{\bf{g}}_r} + {\bf{e}},$$

where ${{\mathbf{g}}_f} \sim \,N\left( {0,{{\mathbf{G}}_f}\sigma _f^2} \right)$ and ${{\mathbf{g}}_r} \sim \,N\left( {0,{{\mathbf{G}}_r}\sigma _r^2} \right)$ represent genetic effects of feature and background SNPs, respectively. Corresponding genomic relationship matrices, ${{\bf{G}}_f}$ and ${{\bf{G}}_r}$, were constructed based on the feature and background SNPs, respectively.

Models involving cis- and trans-eQTL-derived SNPs are designated GFBLUP_cis and GFBLUP_trans, respectively. All BLUP-based models were implemented using the DMU software package [48] for variance component estimation and solution of mixed model equations.

## Results

### Prediction accuracy of gene expression estimated breeding values and its correlation with cis-heritability

**Fig. 2:**
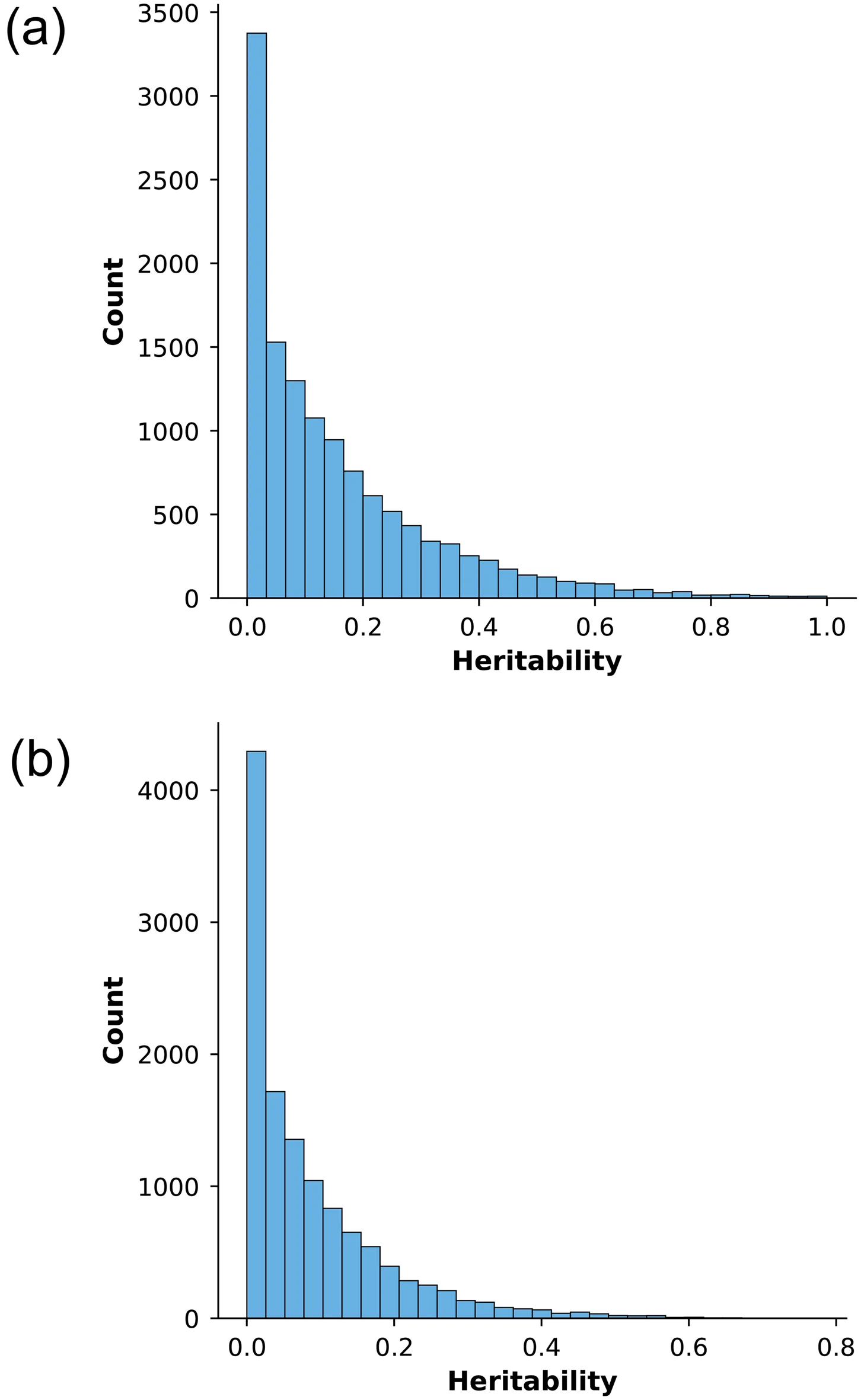
Distribution of cis-heritability estimates for gene expression in scenarios II and III. (**a**) Scenario II; (**b**) Scenario III

We first examined the distribution of cis-heritability estimates for gene expression. In Scenario II, cis-heritability was estimated using the reference population with observed transcriptomes, whereas in Scenario III it was estimated using the ChickenGTEx dataset. In both scenarios, cis-heritability was highly right-skewed, with most genes concentrated at low values and a long tail extending toward higher heritability (Fig. 2). The distribution was slightly more skewed toward low values in Scenario III than in Scenario II. This pattern highlights the sparse architecture of cis-regulatory effects on gene expression and supports the use of heritability filtering in downstream analyses.

Given these cis-heritability distributions, we next evaluated the prediction accuracy (as defined in Materials and methods) of BLUP and ENET for gene expression EBVs predicted from cis-SNPs in Scenarios II and III, stratified by minimum cis-heritability thresholds (Table 1). For each method and threshold, Table 1 reports the average prediction accuracy and *n* = *n*_1_/*n*_2_, where *n*_2_ is the number of genes with successfully predicted EBVs and *n*_1_ is the number of genes with EBV prediction accuracy between −1 and 1 used to calculate the average accuracy. In both scenarios, BLUP successfully fitted all genes included in the analysis. In contrast, ENET successfully predicted EBVs for 9571 genes in Scenario II and 6992 genes in Scenario III when all genes were considered. The difference in the number of successfully fitted genes between BLUP and ENET was largest when no heritability filtering was applied (3110 in Scenario II and 2366 in Scenario III) but decreased markedly after applying the ${h^2} > 0.1$ threshold (275 in Scenario II and 273 in Scenario III) and became very small at higher thresholds. This suggests that the discrepancy in the total number of fitted genes between BLUP and ENET primarily stems from the difference in successful fitting of genes with cis-heritability below 0.1.

**Table 1:** Average prediction accuracy of gene expression EBVs at different cis‑heritability thresholds in scenarios II and III

Scenario	**Model**^**1**^	All	${{\bf{\it{h}}}^2} > 0.$**1**	${{\bf{\it{h}}}^2} > 0.$**3**	${{\bf{\it{h}}}^2} > 0.5$	${{\bf{\it{h}}}^2} > 0.$**7**
II	BLUP	0.386 (*n* = 9543/12,681)^2^	0.470 (*n* = 6167/6408)	0.570 (*n* = 2091/2125)	0.610 (*n* = 674/680)	0.581 (*n* = 186/186)
	ENET	0.401 (*n* = 8133/9571)	0.472 (*n* = 5871/6133)	0.592 (*n* = 2053/2103)	0.641 (*n* = 657/666)	0.624 (*n* = 175/175)
III	BLUP	0.157 (*n* = 7486/9358)	0.207 (*n* = 3134/3161)	0.304 (*n* = 447/452)	0.347 (*n* = 54/55)	0.199 (*n* = 3/3)
	ENET	0.126 (*n* = 5986/6992)	0.180 (*n* = 2864/2888)	0.303 (*n* = 442/446)	0.370 (*n* = 54/55)	0.275 (*n* = 3/3)

^1^BLUP, best linear unbiased prediction; ENET, elastic net. ^2^*n*=*n*_1_/*n*_2_, where *n*_2_ is the number of genes with successfully predicted EBVs and *n*_1_ is the number of genes with EBV prediction accuracy between −1 and 1 used to calculate the average accuracy. BLUP successfully fitted all gene expression EBV prediction models and produced EBVs for all genes, whereas ENET failed to fit some models, and EBVs were not available for those genes. In Scenario III, gene numbers and accuracies were based on genes overlapping between the Tiannong partridge chicken dataset and the ChickenGTEx project dataset

**Fig. 3:**
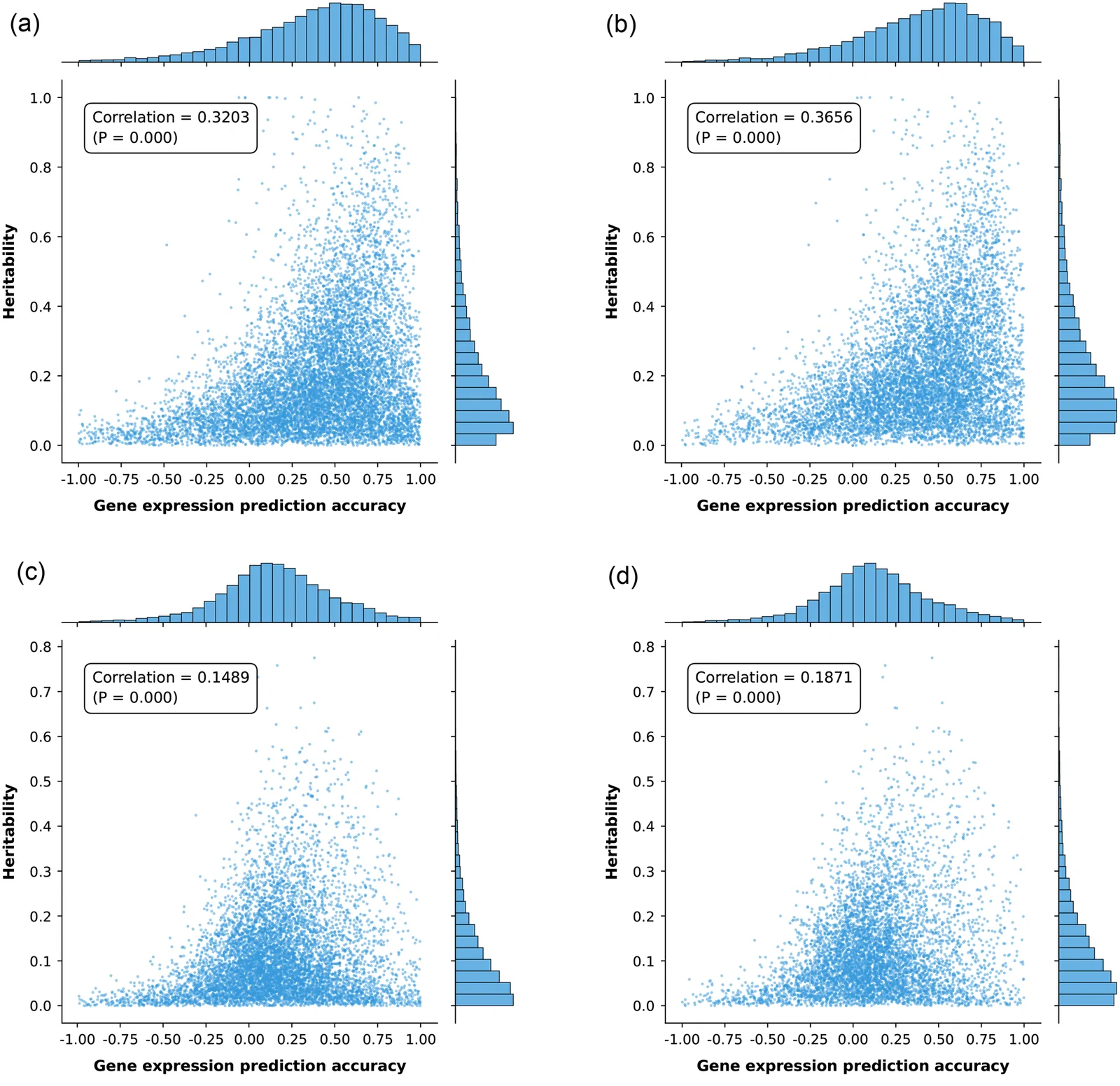
Spearman correlation between cis‑heritability and prediction accuracy of gene expression EBVs in scenarios II and III. (**a**) Scenario II, BLUP; (**b**) Scenario II, ENET; (**c**) Scenario III, BLUP; (**d**) Scenario III, ENET. Correlation data were taken from the 1st fold of the 10th repetition of the five‑fold cross‑validation (out of 10 total repeats). Method abbreviations: BLUP, best linear unbiased prediction; ENET, elastic net

Prediction accuracy of gene expression EBVs was consistently higher in Scenario II than in Scenario III across both methods and thresholds (Table 1). Accuracy generally increased with cis-heritability, but the trend was not strictly monotonic: at the most stringent threshold (${h^2} > 0.7$), accuracy did not exceed that observed at lower thresholds. This likely reflects the very small number of genes retained at this threshold, particularly in Scenario III (3 genes), which increases estimation uncertainty. As quantified in Fig. 3, both BLUP and ENET showed positive correlations between cis-heritability and gene expression EBV prediction accuracy. In Scenario II, the correlations were moderate (Spearman *r* = 0.32 and 0.37 for BLUP and ENET, respectively), whereas in Scenario III the correlation was weak (Spearman *r* = 0.15 and 0.19 for BLUP and ENET, respectively). Overall, cis-heritability was positively associated with EBV–BV accuracy.

### Prediction performance in scenario I: both reference and validation populations have transcriptomic data

**Fig. 4:**
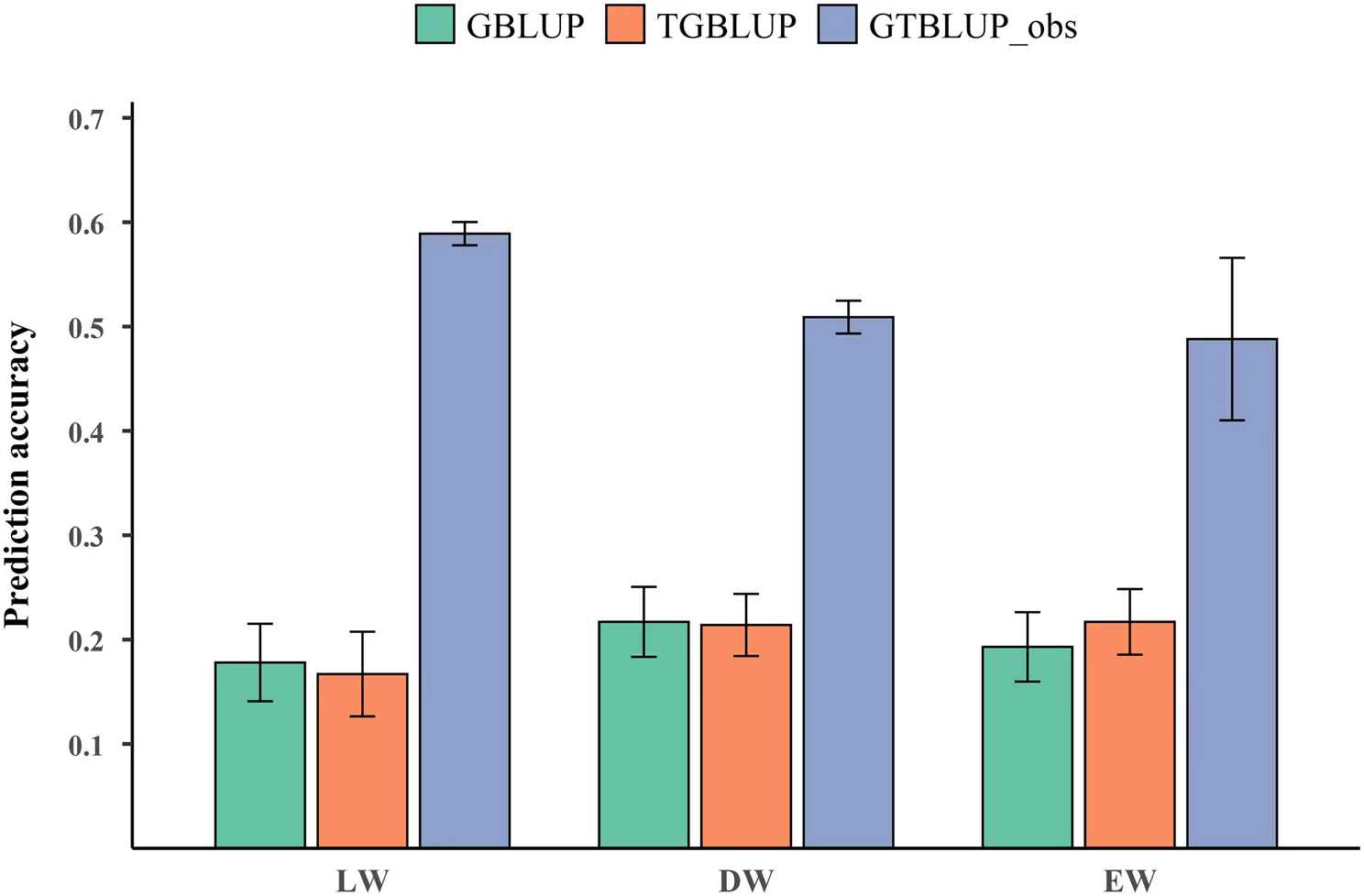
Prediction accuracy of multi-omics genomic prediction methods in Scenario I. Trait abbreviations: LW, live weight; DW, dressed weight; EW, eviscerated weight. Method abbreviations: GBLUP, genomic best linear unbiased prediction; TGBLUP, transcriptomic-genomic best linear unbiased prediction; GTBLUP_obs, genomic-transcriptomic best linear unbiased prediction using observed expression. Error bars represent the standard deviation of prediction accuracies across 10 repeated 5‑fold cross‑validations

As illustrated in Fig. 4, GTBLUP_obs showed the highest prediction accuracies for all three traits. However, GTBLUP_obs uses observed expression, which contains both genetic and environmental components, whereas TGBLUP and GBLUP predict breeding values; therefore, its accuracy is not directly comparable to that of the other methods. Holm-corrected paired *t*-tests revealed that, in this reference comparison, GTBLUP_obs was the only model that significantly outperformed GBLUP, with adjusted *p* = 0.005 for DW, and *p* < 0.0001 for both LW and EW (Additional file 2 Table S1). In contrast, TGBLUP yielded prediction accuracies that were numerically comparable to GBLUP for LW (*r* = 0.167 vs. 0.178) and DW (*r* = 0.214 vs. 0.217), and slightly higher for EW (0.217 vs. 0.193); however, none of these differences were statistically significant after multiple testing correction, with adjusted *p* = 0.80, 0.63, and 0.95 for LW, DW, and EW, respectively (Additional file 2 Table S1).

### Prediction performance in scenario II: validation population lacks transcriptomic data

**Fig. 5:**
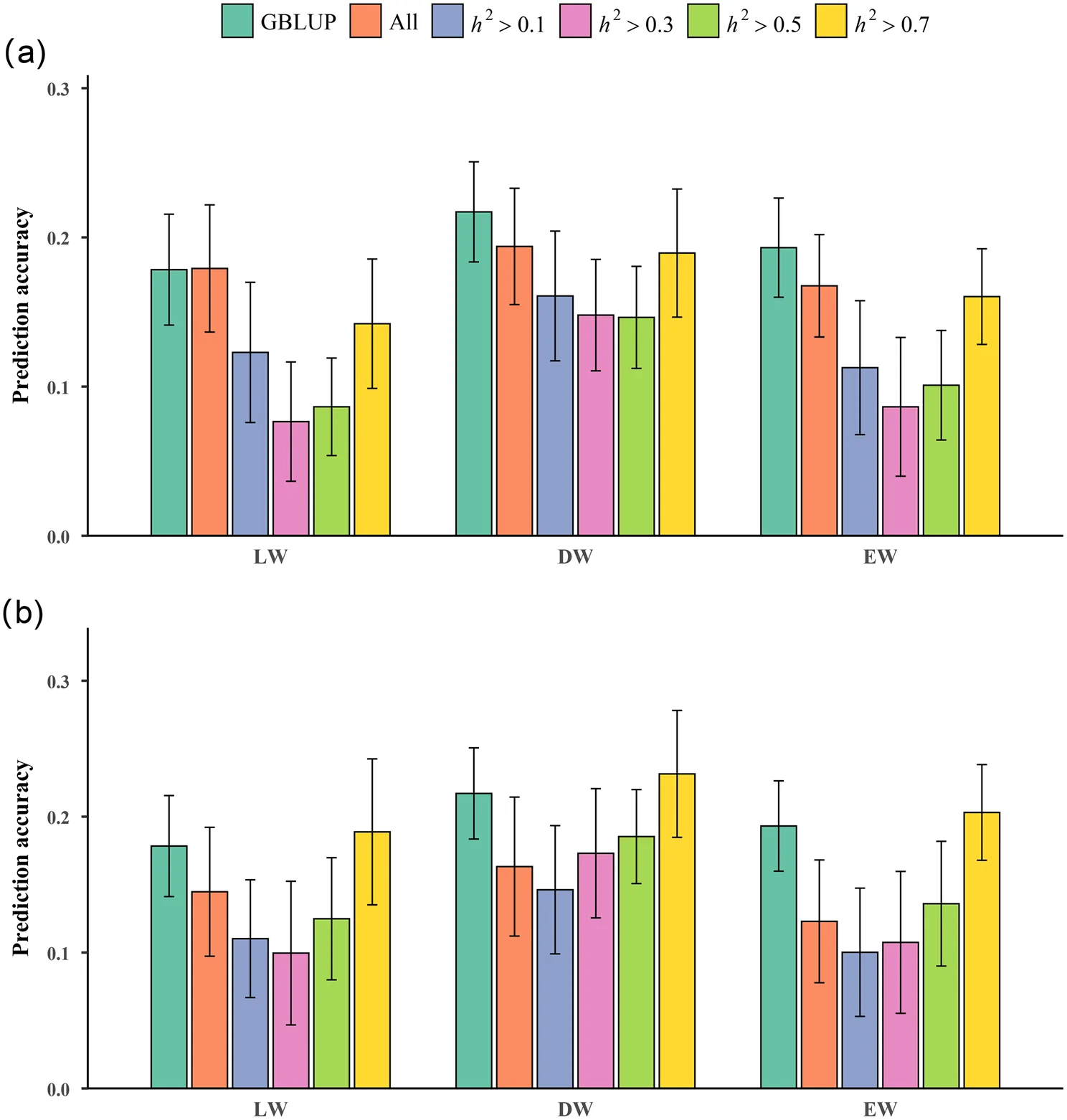
GTBLUP prediction accuracy in Scenario II using predicted expression EBVs of genes selected by heritability thresholds. (**a**) GTBLUP_pred _BLUP; (**b**) GTBLUP_pred _ENET. Trait abbreviations: LW, live weight; DW, dressed weight; EW, eviscerated weight. Method abbreviations: GBLUP, genomic best linear unbiased prediction; GTBLUP_pred_BLUP and GTBLUP_pred_ENET, genomic-transcriptomic best linear unbiased prediction using predicted gene expression estimated breeding values predicted by best linear unbiased prediction or elastic net, respectively. for both GTBLUP_pred_BLUP and GTBLUP_pred_ENET, the notation “*h*^2^ > X” indicates the subset of genes with cis-heritability above the specified threshold (X = 0.1, 0.3, 0.5, or 0.7); “all” denotes the full set of predicted genes. Error bars represent the standard deviation of prediction accuracies across 10 repeated 5‑fold cross‑validations

Figure 5 shows the average genomic prediction accuracies of GTBLUP_pred_BLUP and GTBLUP_pred_ENET under various expression heritability thresholds in Scenario II. Among all configurations, GTBLUP_pred_ENET_0.7 attained the highest numerical accuracies for all traits, with values of 0.189 (LW), 0.231 (DW), and 0.203 (EW)—representing relative gains of 6.2, 6.5, and 5.2% over GBLUP (with accuracies of 0.178, 0.217, and 0.193, respectively). However, none of these differences approached statistical significance after Holm correction, with all adjusted *p* values equal to 1.00 (Additional file 2 Table S2). Similarly, all other GTBLUP_pred_ENET thresholds and all GTBLUP_pred_BLUP configurations generally yielded numerical accuracies that were lower than those of GBLUP for all trait–model combinations; however, all adjusted *p* values were equal to 1.00.

**Fig. 6:**
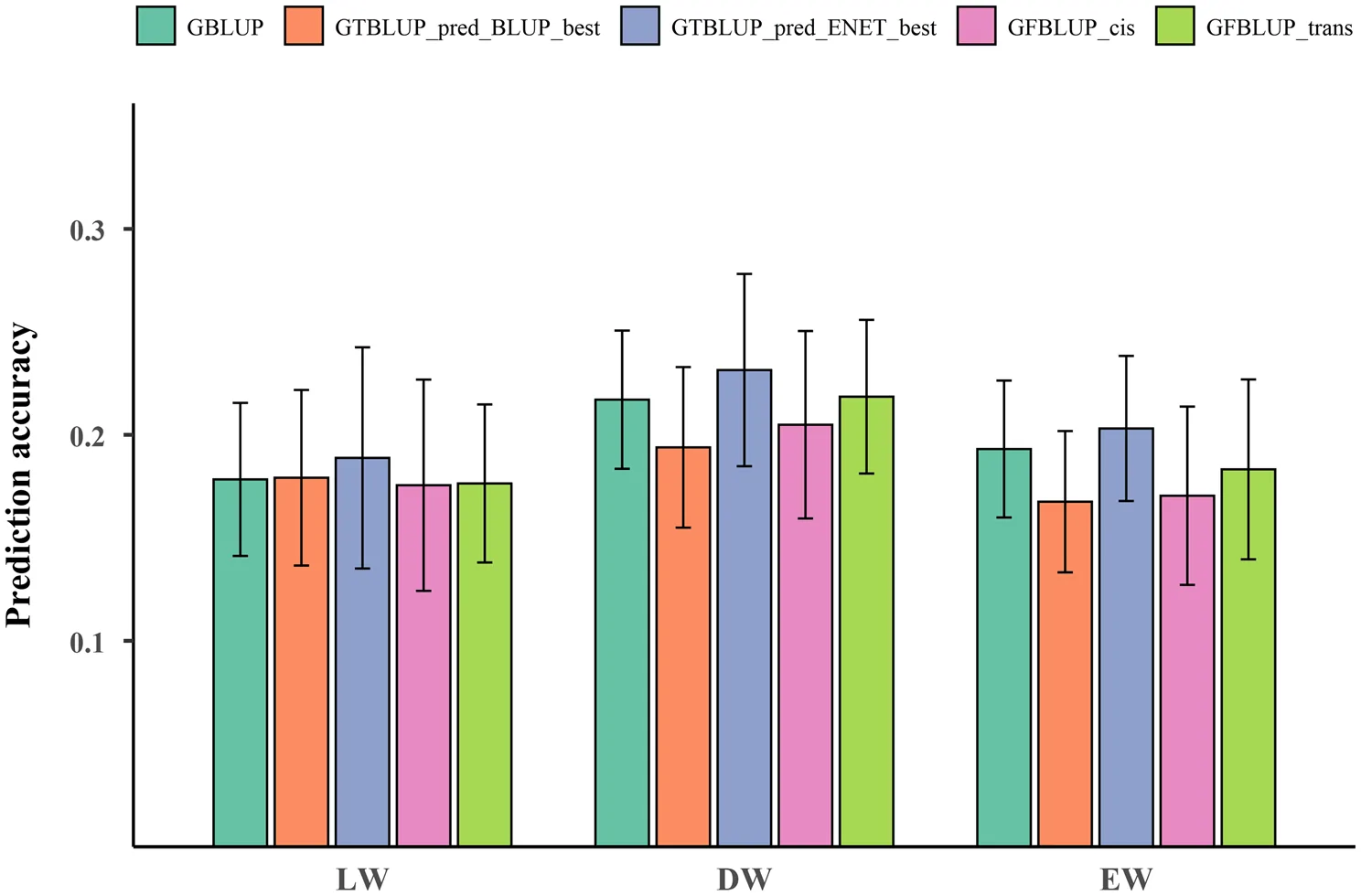
Prediction accuracy of multi-omics genomic prediction methods in Scenario II. Trait abbreviations: LW, live weight; DW, dressed weight; EW, eviscerated weight. Method abbreviations: GBLUP, genomic best linear unbiased prediction. GTBLUP_pred_BLUP_best, the top-performing method among the genomic-transcriptomic best linear unbiased prediction framework, incorporating gene expression estimated breeding values predicted by best linear unbiased prediction. in Scenario II, this corresponds to GTBLUP_pred_BLUP_all (using all predicted genes without cis-heritability filtering). GTBLUP_pred_ENET_best, the top-performing method among the genomic-transcriptomic best linear unbiased prediction framework, incorporating gene expression estimated breeding values predicted by elastic net. in Scenario II, this corresponds to GTBLUP_pred_ENET_0.7 (retaining only genes with cis-heritability > 0.7). GFBLUP_cis and GFBLUP_trans, genomic-feature best linear unbiased prediction models incorporating SNPs derived from cis-eQtls and trans-eQtls, respectively. Error bars represent the standard deviation of prediction accuracies across 10 repeated 5‑fold cross‑validations

As depicted in Fig. 6, GTBLUP_pred_ENET_best (i.e., GTBLUP_pred_ENET_0.7) exhibited the most favorable numerical profile among all models across the three traits. However, its accuracy did not differ significantly from that of GBLUP for any of the three traits, with all adjusted *p* values equal to 1.00 (Additional file 2 Table S2). GTBLUP_pred_BLUP_best had numerically slightly higher accuracies for LW but lower for DW and EW—though all adjusted *p* values were 1.00. Among the feature screening models, GFBLUP_trans produced a marginal numerical gain in accuracy for DW (+0.7% relative to GBLUP), yet again without statistical support (adjusted *p* = 1.00).

### Prediction performance in scenario III: neither reference nor validation population has transcriptomic data

**Fig. 7:**
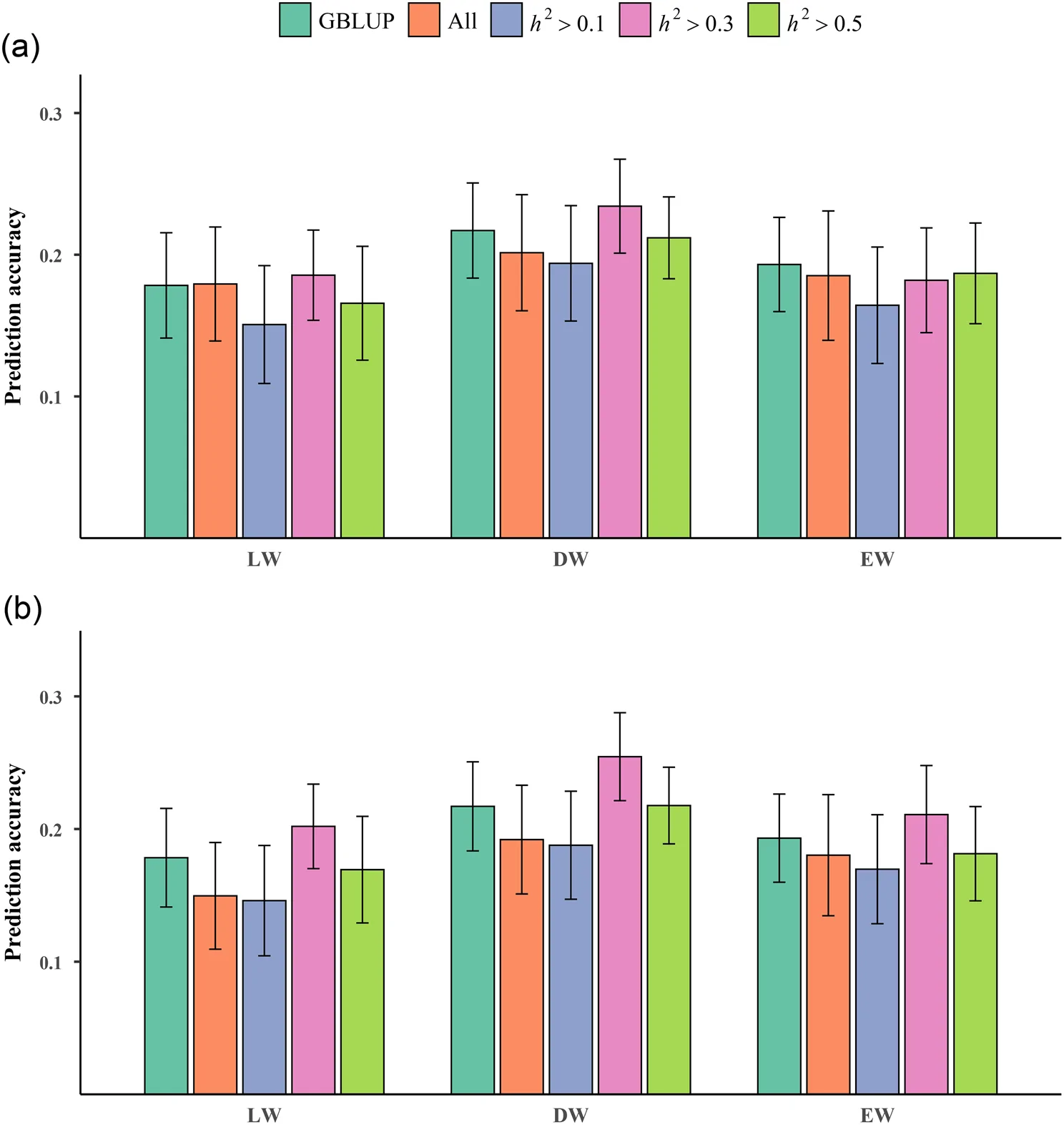
GTBLUP prediction accuracy in Scenario III using predicted expression EBVs of genes selected by heritability thresholds. (**a**) GTBLUP_pred _BLUP; (**b**) GTBLUP_pred _ENET. Trait abbreviations: LW, live weight; DW, dressed weight; EW, eviscerated weight.Method abbreviations: GBLUP, genomic best linear unbiased prediction; GTBLUP_pred_BLUP and GTBLUP_pred_ENET, genomic-transcriptomic best linear unbiased prediction using gene expression estimated breeding values predicted by best linear unbiased prediction or elastic net, respectively. for both GTBLUP_pred_BLUP and GTBLUP_pred_ENET, the notation “*h*^2^ > X” indicates the subset of genes with cis-heritability above the specified threshold (X = 0.1, 0.3, 0.5, or 0.7); “all” denotes the full set of predicted genes. Error bars represent the standard deviation of prediction accuracies across 10 repeated 5‑fold cross‑validations

Figure 7 shows the average genomic prediction accuracies of GTBLUP_ pred_BLUP and GTBLUP_ pred_ENET under different expression heritability thresholds in Scenario III. Despite using the ChickenGTEx dataset solely for training gene expression EBV prediction models (without population-specific transcriptomic data), both GTBLUP_ pred_ENET and GTBLUP_ pred_BLUP (except for EW) produced numerical accuracies greater than those of GBLUP when the heritability threshold was set at ${h^2} > 0.3.$ Notably, GTBLUP_pred_ENET_0.3 yielded the largest numerical gains, with relative increases of 13.5, 17.5, and 9.3% over GBLUP for LW, DW, and EW, respectively. GTBLUP_pred_BLUP_0.3 also showed numerical improvements of 4.5 and 7.8% for LW and DW. However, none of these observed differences reached statistical significance after Holm correction, with all adjusted *p* values equal to 1.00 (Additional file 2 Table S3).

**Fig. 8:**
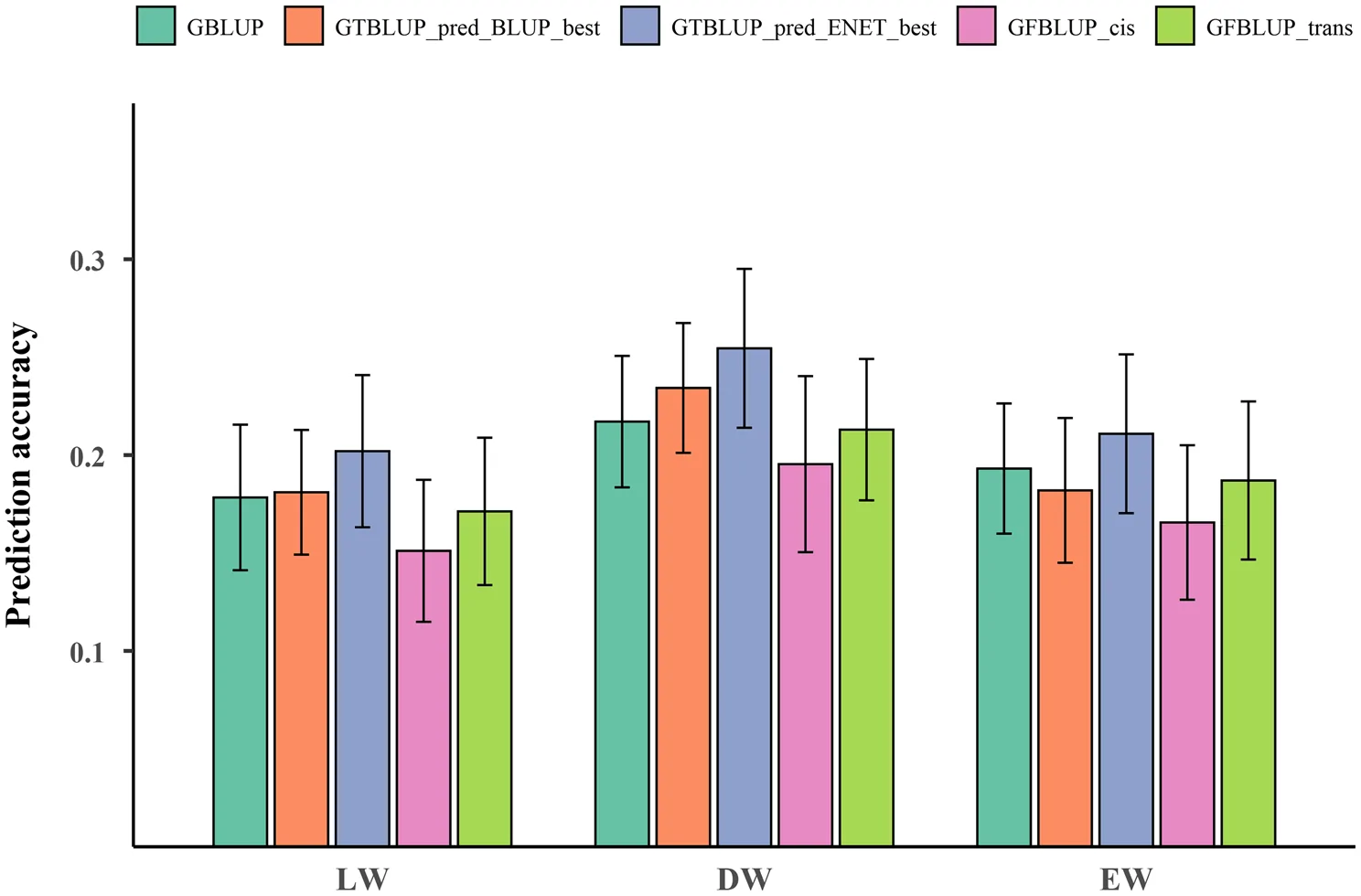
Prediction accuracy of multi-omics genomic prediction methods in Scenario III. Trait abbreviations: LW, live weight; DW, dressed weight; EW, eviscerated weight. Method abbreviations: GBLUP, genomic best linear unbiased prediction. GTBLUP_pred_BLUP_best, the top-performing method among the genomic-transcriptomic best linear unbiased prediction framework, incorporating gene expression estimated breeding values predicted by best linear unbiased prediction. in Scenario III, this corresponds to GTBLUP_BLUP_0.3 (retaining only genes with cis-heritability > 0.3) for LW and DW, and GTBLUP_BLUP_0.5 (retaining only genes with cis-heritability > 0.5) for EW. GTBLUP_pred_ENET_best, the top-performing method among the genomic-transcriptomic best linear unbiased prediction framework, incorporating gene expression estimated breeding values predicted by elastic net. in Scenario III, this corresponds to GTBLUP_pred_ENET_0.3 (retaining only genes with cis-heritability > 0.3). GFBLUP_cis and GFBLUP_trans, genomic-feature best linear unbiased prediction models incorporating SNPs derived from cis-eQtls and trans-eQtls, respectively. Error bars represent the standard deviation of prediction accuracies across 10 repeated 5‑fold cross‑validations

Figure 8 presents genomic prediction accuracies of all models in Scenario III. Model GTBLUP_pred_ENET_best (i.e., GTBLUP_pred_ENET_0.3) achieved the highest numerical accuracy overall, surpassing all other evaluated models for all three traits. However, none of its observed advantages over GBLUP reached statistical significance after Holm correction (adjusted *p* = 1.00), consistent with the results for all other models in this scenario (Additional file 2 Table S3). Notably, the eQTL-derived feature selection models—GFBLUP_cis and GFBLUP_trans—performed numerically worse than GBLUP for all three traits, further highlighting the limited utility of transferring regulatory information from external reference datasets when population-specific transcriptomic data are not available in both reference and validation populations.

## Discussion

The primary objective of this study was to assess whether gene expression EBVs predicted solely from genotype data can enhance genomic prediction accuracy in breeding programs where RNA-seq profiling in the target population is not available or impractical. To address this, we adapted BLUP and ENET for predicting cis-expression EBVs from genotypes and integrated these predicted EBVs into an extended GTBLUP framework to estimate GEBVs. Critically, this strategy circumvents the need for observed transcriptomes in the target population—a key limitation of existing methods such as GTBLUP with observed expression (GTBLUP_obs) and TGBLUP, which require expression measurements in both reference and target populations and were therefore only applicable in Scenario I. Across two realistic breeding scenarios (II and III), where expression was absent in the target set, GTBLUP with predicted gene expression EBVs yielded accuracies that were numerically comparable to or greater than those of standard GBLUP; however, none of these observed differences reached statistical significance after Holm correction (all adjusted *p* = 1.00). These findings indicate that while predicted gene expression EBVs shows promise, its practical utility relative to GBLUP warrants further investigation with larger reference populations or refined prediction models.

In Scenario I, where observed gene expression data were available on the target population, GTBLUP_obs incorporates observed expression levels that capture not only additive genetic effects, but also environmental influences and potential non-additive contributions. As a result, the predicted outcome more closely predicts the phenotypic value rather than the true breeding value [41, 49]. This explains why GTBLUP_obs achieved the highest prediction accuracy in this scenario—because it benefits from all sources of variation that are correlated with the phenotype, including the prediction errors of the EBV. Holm-corrected paired *t*-tests confirmed that GTBLUP_obs was the only model that significantly outperformed GBLUP (adjusted *p* < 0.0001 for LW and EW; adjusted *p* = 0.005 for DW). Although it cannot be used in practical breeding programs, GTBLUP_obs provides a theoretical upper bound on prediction accuracy, serving as a benchmark for evaluating GTBLUP_pred_BLUP and GTBLUP_pred_ENET in Scenarios II and III. These latter models instead use predicted gene expression EBVs—derived from either BLUP or ENET models trained on SNP genotypes—which aim to extract the heritable, cis-regulated component of transcript abundance [19]. By relying on these EBVs of gene expression, GTBLUP_pred_BLUP and GTBLUP_pred_ENET effectively model additive genetic contributions and produce predictions that are interpretable as breeding values.

Similarly, TGBLUP—also evaluated exclusively in Scenario I—adopts a two-step approach: first estimating effects of both genomic markers and observed gene expressions, then partitioning the additive genetic component from the transcriptomic effects in the second step. The final prediction combines genomic and transcriptomic additive effects into an integrated estimate of total breeding value, aligning well with the objectives of genetic evaluation and selection. For comparison, GBLUP and GFBLUP rely solely on genomic marker data to capture additive genetic variation, and their predictions are, therefore, standard estimates of breeding values. Therefore, except for GTBLUP_obs, all other models evaluated—GBLUP, GFBLUP, GTBLUP_pred_BLUP, GTBLUP_pred_ENET, and TGBLUP—produce predictions that represent estimates of breeding values, making them comparable across methods and directly suitable for implementation in genetic improvement programs.

In Scenario II, although not statistically significant, the GTBLUP_pred_ENET_0.7 model—incorporating only genes with predicted expression heritability ${h^2} > 0.7$—showed the highest numerical genomic prediction accuracies, corroborating findings by Wang et al. [41]. This observation also aligns with earlier work demonstrating that gene expression EBVs must reach a certain accuracy threshold (e.g., correlation > 0.55) to significantly enhance genomic prediction [18]. Our results further support the low-to-moderate positive relationship between cis-heritability of gene expression and prediction accuracy (Fig. 3), suggesting that prioritizing genes with high heritability enhances model performance.

Interestingly, in Scenario III, filtering genes based on high heritability led to reduced genomic prediction accuracy numerically, where selecting genes with ${h^2} > 0.5$ resulted in lower accuracy than using those with ${h^2} > 0.3$. This suggests that overly stringent filtering can lead to information loss, especially when prediction accuracy across genes is already high. Reducing the number of integrated expression EBVs increases model reliance on a limited subset of transcripts, potentially compromising robustness and generalizability [10, 50]. Therefore, optimal performance likely depends on balancing the inclusion of informative, high-quality expression EBVs, while avoiding excessive sparsity in the omics layer [12, 18, 41, 51].

Several factors are known to influence the prediction accuracy of gene expression EBVs, including the choice of statistical method, training sample size, genetic similarity between training and target populations, the choice of SNP markers used for prediction, and tissue specificity and developmental timing of omics measurements [20, 40]. These factors are equally critical for multi‑omics genomic prediction, because the accuracy of predicted expression EBVs directly affects the subsequent prediction of breeding values for the trait, and additionally, the choice of multi‑omics integration model itself also influences prediction performance. In our study, we predicted expression EBVs using BLUP and ENET, and ENET generally showed numerically higher performance than BLUP in Scenarios II and III. The classical BLUP approach assumes polygenic inheritance and effectively captures additive genetic signals using cis-SNPs to construct the genomic relationship matrix [6]. ENET, in contrast, applies L_1_ and L_2_ penalties to perform variable selection and shrinkage, which can be advantageous for modeling sparse or complex regulatory architectures [52], particularly when prediction must be generalized across populations or conditions. However, similar variable selection can also be achieved by Bayesian variable-selection approaches such as Bayes-B, which uses a sparse mixture prior to select key regulatory variants [1]. Because our study compared only BLUP and ENET, we do not claim that ENET is uniquely or generally superior to Bayesian variable-selection methods. Future work should therefore focus on optimizing existing models—or developing new ones—specifically tailored to improve gene expression EBV prediction accuracy for low-heritability genes and in cross-population settings.

Additionally, the choice of multi-omics integration model—whether additive (e.g., GTBLUP), hierarchical (e.g., Bayesian multitask learning), or nonlinear (e.g., deep neural networks)—directly influences the accuracy of multi-omics genomic prediction, as each approach embodies distinct assumptions about biological data dependencies and noise structures. While the present study adopted an extended GBLUP framework (GTBLUP) for integrative prediction, future work should prioritize modeling strategies that explicitly account for the hierarchical and conditional nature of multi-omics integration—such as hierarchical Bayesian models that jointly infer SNP effects, expression regulation, and phenotypic outcomes, or interpretable deep learning architectures designed for cross-omics alignment.

In this study, we restricted the analysis to common cis-SNPs shared between datasets, which inevitably excluded potentially informative variants and reduced model power. Moreover, the exclusion of trans-acting variants—a consequence of focusing solely on cis-SNPs—likely limited the explanatory power of the expression EBV prediction models [53]. Given that trans-regulatory effects play a significant role in gene regulation, future efforts should explore the incorporation of genome-wide SNPs or prioritize biologically informed sets of trans-eQTL [54, 55] to further improve the prediction accuracy of gene expression EBVs.

To ensure biological relevance and consistency across scenarios, we selected muscle tissue as the source of transcriptomic data for cis-regulatory gene expression EBV prediction in Scenario III. This choice aligns directly with the tissue used in Scenario II and reflects the well-established physiological role of muscle in determining the target traits: body weight, carcass weight, and eviscerated weight. Muscle is not only a major component of carcass mass but also a key metabolic and contractile tissue whose gene expression profiles are highly correlated with growth efficiency, protein deposition, and meat yield in poultry [56, 57]. From a practical standpoint, among various tissues profiled in ChickenGTEx, muscle had 517 high-quality RNA-seq samples—the second largest sample size after liver (*n* = 749). Despite this relatively large sample size, the use of the ChickenGTEx dataset in Scenario III yielded lower gene expression EBV prediction accuracy compared to within‑population training. This outcome reflects well-documented challenges in cross-population genomic prediction, including differences in linkage disequilibrium patterns, allele frequencies, and technical variability in sample processing [40], and genotype-by-environment (G×E) interactions that can shape gene expression in a context-dependent manner [58]. As FarmGTEx and related efforts continue to expand the scale, breed diversity, and standardization of publicly available livestock transcriptomic resources, the accuracy and generalizability of transcriptome-based prediction of gene expression EBVs are expected to improve substantially.

It is important to acknowledge that the relatively small reference population size (*n* = 350)—typical of many studies focused on local or conservation-oriented poultry populations—constrains both the statistical power to detect subtle but biologically meaningful differences among prediction models and the asymptotic accuracy ceiling of conventional GBLUP. Under such conditions, the realized accuracy of GBLUP is likely attenuated, which may inflate the relative numerical advantage observed for GTBLUP in this study. Indeed, under both Scenarios II and III, despite numerically higher accuracy for the best‑performing GTBLUP model in all comparisons, none of these differences reached statistical significance with the current sample size (all adjusted *p* = 1.00), reflecting the limited statistical power noted above. Furthermore, formal pairwise comparisons of prediction accuracy between Scenarios II and III—using the repkfold_ttest() function from the R package correctR [38], which accounts for the non-independence of repeated k-fold cross-validation estimates—yielded no statistically significant difference (*p* > 0.05).

In future work, we will assess model performance using large-scale genomic and transcriptomic datasets from additional livestock species. In particular, systematic comparisons across varying reference population sizes are needed to examine whether the gain from incorporating gene expression EBVs diminishes as the sample size increases. Accordingly, we will quantify the cost–benefit trade-off: weighing the experimental costs of RNA-seq (or imputation infrastructure) and computational overhead against gains in prediction accuracy. Such evidence-based evaluation is essential to determine whether, and under what conditions, multi-omics integration transitions from a methodological innovation to an economically sustainable component of routine genomic prediction in livestock breeding programs.

## Conclusions

In this study, only GTBLUP with observed transcriptomes (Scenario I) achieved statistically significant superiority in predicting trait phenotypes over GBLUP (Holm-adjusted *p* < 0.05). In scenarios lacking target-population transcriptomic data, the best-performing GTBLUP configurations—particularly those using ENET-predicted gene expression EBVs with appropriate cis-heritability filtering—yielded numerically higher accuracies than GBLUP and eQTL-based GFBLUP; however, none of these advantages reached significance after multiple-testing correction. The numerical gains were modulated by prediction reliability and heritability, with excessive filtering diminishing performance, and models using ENET-derived gene expression EBVs numerically outperformed those using BLUP-derived ones. GFBLUP with eQTL-based feature selection showed limited effectiveness. Collectively, predicted gene expression EBVs show promise as a complementary biological prior, but the practical advantage of integrating these predicted EBVs over conventional GBLUP remains numerically driven and statistically unsubstantiated given the relatively small reference populations that were used here, warranting further validation in larger cohorts.

## Electronic supplementary material

Below is the link to the electronic supplementary material.

Supplementary Material 1

Supplementary Material 2

## Data Availability

The datasets used during the present study are available from the corresponding author on reasonable request.
