# Supplementary material for: Integrating predicted gene expression into genomic selection: an evaluation in partridge chickens under realistic breeding scenarios
Source: Genet Sel Evol. 2026 Nov 9;58:83. doi: 10.1186/s12711-026-01099-w (PMC13746541; doi:10.1186/s12711-026-01099-w)
Supplement: Supplementary file 1 [file 12711_2026_1099_MOESM2_ESM.pdf]

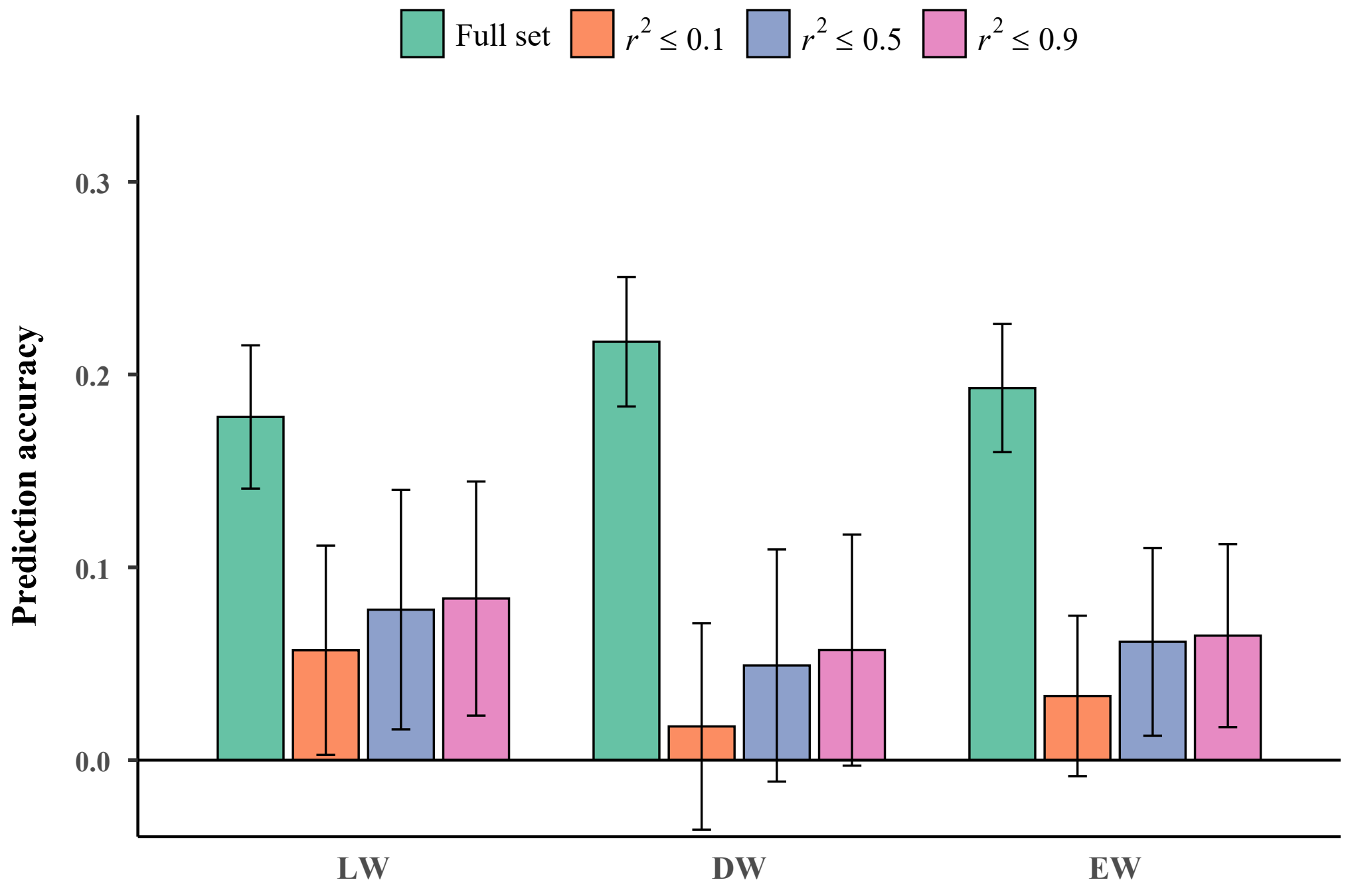

Full set
$r^2 \leq 0.1$
$r^2 \leq 0.5$
$r^2 \leq 0.9$
Prediction accuracy
0.3
0.2
0.1
0.0
LW
DW
EW
